# TGA/Chemometric Test Is Able to Detect the Presence of a Rare Hemoglobin Variant Hb Bibba

**DOI:** 10.3389/fmolb.2019.00101

**Published:** 2019-10-01

**Authors:** Roberta Risoluti, Patrizia Caprari, Giuseppina Gullifa, Loretta Diana, Matteo Luciani, Antonio Amato, Stefano Materazzi

**Affiliations:** ^1^Department of Chemistry, Sapienza University of Rome, Rome, Italy; ^2^National Centre for the Control and Evaluation of Medicine, Istituto Superiore di Sanità, Rome, Italy; ^3^UO Ematologia, Ospedale Pediatrico Bambino Gesù, Rome, Italy; ^4^ANMI Onlus, Centro Studi Microcitemie, Rome, Italy

**Keywords:** thermogravimetric analysis, chemometrics, Hb Bibba, hemoglobin defect, screening

## Abstract

In this study the TGA/Chemometric test was applied for diagnosis of a case of congenital hemolytic anemia for which the common first level diagnostic tests were not able to find the erythrocyte congenital defect. A 6 years old girl presented chronic hemolytic anemia characterized by hyperbilirubinemia, increased spleen, negative Coombs tests, normal hemoglobin values, decreased mean corpuscular volume (MCV), increased red cell distribution width (RDW), reticulocytes and lactate dehydrogenase (LDH), and altered erythrocyte morphology (ovalocytes, spherocytes, and rare schizocytes). The diagnostic protocols for differential diagnosis of hereditary hemolytic anemia were carried out by the investigation of the congenital hemolytic anemias due to defects of membrane proteins and the most common erythrocyte enzymes, but no defect was found. The TGA/Chemometric test was applied and the PLS-DA model of prediction was used to process results. The thermogravimetric profile of the patient was very distinct from those of healthy subjects and comparable with those of thalassemia patients. The classification model applied to the patient identified a chronic hemolytic anemia due to a hemoglobin defect and the molecular characterization confirmed the TGA/Chemometrics results, demonstrating the presence of a very rare hemoglobin variant Hb Bibba (α_2_136(H19)Leu → Proβ_2_). In conclusion the TGA/Chemometric test proved to be a promising tool for the screening of the hemoglobin defects, in a short time and at low cost, of this case of congenital hemolytic anemia of difficult diagnosis. This method results particularly suitable in pediatric patients as it requires small sample volumes and is able to characterize patients subjected to transfusion.

## Introduction

The differential diagnosis of hereditary hemolytic anemias (Haley, 2017) is generally carried out by applying different diagnostic protocols depending on the specific congenital erythrocyte defects such as hereditary erythrocyte enzyme deficiencies (Grace and Glader, 2018), RBC membrane proteins defects (King and Zanella, 2013), or hemoglobinopathies (Cao and Galanello, 2010).

Hemoglobin disorders are characterized by pathologic defects on globin chain synthesis: quantitative defects that give rise to thalassemia (mainly α and β thalassemia); qualitative defects, namely hemoglobinopathies, that are due to structural hemoglobin variants; hereditary persistence of fetal hemoglobin. These globin defects determine a wide array of heterogeneous thalassemia syndromes and related diseases. The diagnosis of hemoglobinopathy could be particularly difficult in neonatal period, especially when transfusions are needed to compensate anemia. Generally, the protocol for hemoglobinopathy screening includes the whole blood count followed by the determination of hemoglobin A_2_ (HbA_2_) and hemoglobin F (HbF) levels, as well as the identification of any rare variant present by Hb electrophoresis or high performance liquid chromatography. Nevertheless, there do exist silent β thalassemic mutations with normal HbA_2_ levels or borderline elevated HbA_2_ values of difficult interpretation (Weatherall and Clegg, 2001). These methods need equipment, time and personnel with expertise in the interpretation of the screening results. A positive screening test still needs to be confirmed by molecular analysis of the globin genes (Cao et al., 2002; Giardine et al., 2014).

Thermoanalytical techniques such as thermogravimetry, proved to be versatile tools able to investigate different materials (Di Donna et al., 2004; De Angelis Curtis et al., 2008; Skreiberg et al., 2011; Fonseca et al., 2012; Materazzi and Risoluti, 2014; Materazzi et al., 2014c; Aiello et al., 2015; Shan-Yang et al., 2015; Papadopoulos et al., 2016; Marcilla et al., 2017; Risoluti et al., 2017). In addition, hyphenated techniques based on thermogravimetry demonstrated the improvement in the ability of this approach to further characterize samples for different applications (Materazzi et al., 2014a,b, 2015; Risoluti et al., 2016c).

Recently, the feasibility of thermogravimetry coupled to chemometrics in processing complex matrices is emerging as rapid and effective tool allowing the multiparametric analysis of different samples (Khanmohammadi et al., 2012; Caramés-Alfaya et al., 2013; Strzemieck et al., 2014). The main advantage of this approach consists of the possibility to identify significant and diagnostic differences in the data and to simultaneously correlate results from different measurements. Therefore, thermoanalytical data obtained from the TGA may be dependent on more than one variable simultaneously (corresponding to the different increase in temperature) or may be a results of specific experimental design where variables such as the concentration, pH, polymer, or metal ligands are randomizing modified to assess the most performing results. In both cases, a chemometric approach based on multivariate analysis becomes very useful as the nature of data is multivariate.

Regardless to clinical application, thermogravimetry coupled with chemometrics demonstrated to be an effective diagnostic tool for β-thalassemia screening requiring short times and low costs of analysis (Risoluti et al., 2016b). This model, consisting of Partial Least Square-Discriminant Analysis (PLS-DA), permitted the discrimination of thalassemic patients and healthy individuals, using the thermogravimetric curves of blood samples. In addition, the TGA screening test allowed differentiating thalassemia patients according to disease clinical severity and was not influenced by drug therapies, such as aspirin, commonly used to prevent thromboembolic events in thalassemia patients after splenectomy (Romolo et al., 2015; Catauro et al., 2018; Risoluti et al., 2018a,b, 2019).

The objective of this study was the application of the TGA/Chemometric test, previously used for thalassemia diagnosis, to investigate a rare case of chronic hemolytic anemia of difficult interpretation, for which the first level tests of the conventional diagnostic protocols were not able to find the erythrocyte congenital defect.

## Materials and Methods

### Blood Samples

In this study we have analyzed blood samples from β-thalassemia patients (65 subjects) and healthy individuals (120 subjects) that were obtained according to guidelines established by the Ethical Committee for human subject studies, in accordance with the 1975 Helsinki Declaration, revised in 2008. All the participants provided their written informed consent to participate in this study and copies of the informed consent are available on request. In the case of the child, the written informed consent was obtained from the parents.

### Patient

A 6-year-old Italian girl, followed at the U.O. Hematology of the Bambino Gesù Pediatric Hospital of Rome (Italy), was suffering from chronic hemolytic anemia of a nature not determined. The clinical picture was characterized by anemia, hyperbilirubinemia, and an increased spleen volume. Laboratory tests demonstrated negative direct and indirect Coombs tests, and increased LDH value that suggested the presence of an erythrocyte congenital defect as cause of the hemolytic anemia. Therefore, investigations have been carried out for the screening of congenital hemolytic anemias from hemoglobin disorders, defects of membrane proteins and the most common red blood cell enzymopathies.

### Hematological Analyses

Blood samples were collected in K_2_EDTA and the hematological parameters red blood cell counts (RBC), Hb, MCV, mean corpuscular hemoglobin (MCH), mean corpuscular hemoglobin concentration (MCHC) and RDW, reticulocyte counts (Ret) were measured by an automated hematology analyzer (ADVIA 120; Siemens, USA). Laboratory examinations also included the evaluation of red cell morphology on peripheral blood smears, the determination of bilirubin, serum ferritin, haptoglobin, transferrin, hemoglobin fractions Hb A_2_, Hb F, Hb S, and Hb C, osmotic fragility test, acidified glycerol lysis time (AGLT_50_) (King and Zanella, 2013) and the activities of erythrocyte enzymes glucose-6-phospate dehydrogenase (G6PD), pyruvate kinase (PK), esokinase (HK), 6-phosphogluconate dehydrogenase (6PGD) (Caprari et al., 1991). The membrane proteins analysis was performed by SDS-PAGE of erythrocyte membranes (Caprari et al., 1999).

### Genetic Analysis for Globin Mutations

Molecular analysis of DNA sequences of β and α globin genes were carried out, at the Microcitemie Center in Rome, by direct sequencing on the Beckman Coulter CEQTM8000 Genetic Analysis System (Beckman Coulter Inc., Fullerton, CA, USA) as previously described (Amato et al., 2012).

### Thermogravimetric Analysis (TGA)

A Perkin Elmer TGA7 Thermobalance (Massachusetts, USA) was used to acquire the thermogravimetric curves. About 30 μl of whole blood was placed into the crucible with no pretreatment. Temperature was measured using a thermocouple directly attached to the crucible and was raised from 20 to 800°C, with a 10°C/min heating rate, as the best resolution rate. The atmosphere was air as carrier gas at 100 ml/min flow rate. Calibration of the thermobalance was performed using the Curie-point transition of standard metals, as specified by the equipment recommendations and a number of three replicates for each sample were acquired to ensure reproducibility. Derivative Thermogravimetric data (DTG) were also calculated to compare samples and represent the derivative of the function TG(T) with respect T.

### Analytical Strategy and Chemometrics

Chemometrics was used to process the thermogravimetric curve of the anemic patient and to compare results of the mass changes as a function of temperature with those of healthy and thalassemia subjects from the collected dataset.

Multivariate statistical analysis based on Principal Component Analysis (PCA; Risoluti et al., 2016a; Materazzi et al., 2017c) was used as exploratory method, while Partial Least Square Linear Discriminant Analysis (PLS-DA; Savitzky and Golay, 1964; Barker and Rayens, 2003; Materazzi et al., 2017a,b) was applied as the classification model of prediction. Each blood sample was analyzed three times and data was reported as means ± standard deviations. The Pyris software (Thermo Fisher Scientific Inc., Waltham, MA, USA) was utilized for diagnostics and acquisition of the thermogravimetric curves and data were exported as ASCII files. The Unscrambler package by Camo was used to perform statistical analysis.

## Results

### Screening Tests for Congenital Hemolytic Anemias

The diagnostic protocols for differential diagnosis of congenital hemolytic anemia were applied and the results are reported in Table 1. The hematological data showed the presence of hemolytic anemia as demonstrated by the increase in total and indirect bilirubin, and LDH values and the low haptoglobin level. The full blood count revealed a decrease in the RBC, Hb, MCV, and MCH values, an increase in the RDW value and reticulocytes count, while the peripheral blood smear showed anisopoikilocytosis and the presence of ovalocytes, spherocytes, schistocytes, and dacryocytes suggesting congenital alterations of erythrocyte morphology. The application of the screening tests for spherocytic hemolytic anemia did not evidenced osmotic fragility of the erythrocytes (Table 1) and the study of erythrocyte membrane proteins did not detect qualitative and quantitative abnormalities of the membrane proteins (data not showed). The screening tests for thalassemia and hemoglobinopathies demonstrated normal HbA_2_ and HbF values, no presence of other hemoglobin variants such as Hb C or Hb S, therefore the presence of hemoglobinopathy was excluded. The study of the enzymatic activities of red blood cell metabolism did not show enzyme defects, while the erythrocyte activities were increased consistent with a chronic hemolytic state and reticulocytosis (Table 1).

**Table 1:** Hematological data of the patient.

	**Value**	**Reference intervals**
RBC (10^∧^6/μL)	3.92	3.8–4.8
Hb (g/dL)	8.8	10.5–15.5
Hct (%)	30.4	33–40
MCV (fL)	73.5	75–95
MCH (pg)	22.4	25–35
MCHC (g/dL)	29.0	31–36
RDW (%)	21.8	12–15
Reticulocyte (%)	9.9	0.2–2.0
Total bilirubin (mg/dL)	2.75	0.25–1.00
Direct bilirubin (mg/dL)	1.08	0.08–1.00
Haptoglobin (mg/dL)	1	30–200
LDH (IU/L)	688	230–470
Transferrin (mg/dL)	241	200–365
Ferritin (mg/dL)	56	10–290
Hb A_2_ (%)	2.5	2.0–3.2
HB F (%)	0.9	0–2.0
Hb S (%)	Absent	Absent
Hb C (%)	Absent	Absent
AGLT_50_ (s)	>1800	>1800
G6PD (IU/g Hb)	16.0	7.0–9.6
6PGD (IU/g Hb)	16.4	7.7–9.6
PK (IU/g Hb)	35	12.0–16.3
HK (IU/g Hb)	3.0	0.8–1.4

### TGA/Chemometric Test

**Figure 1:**
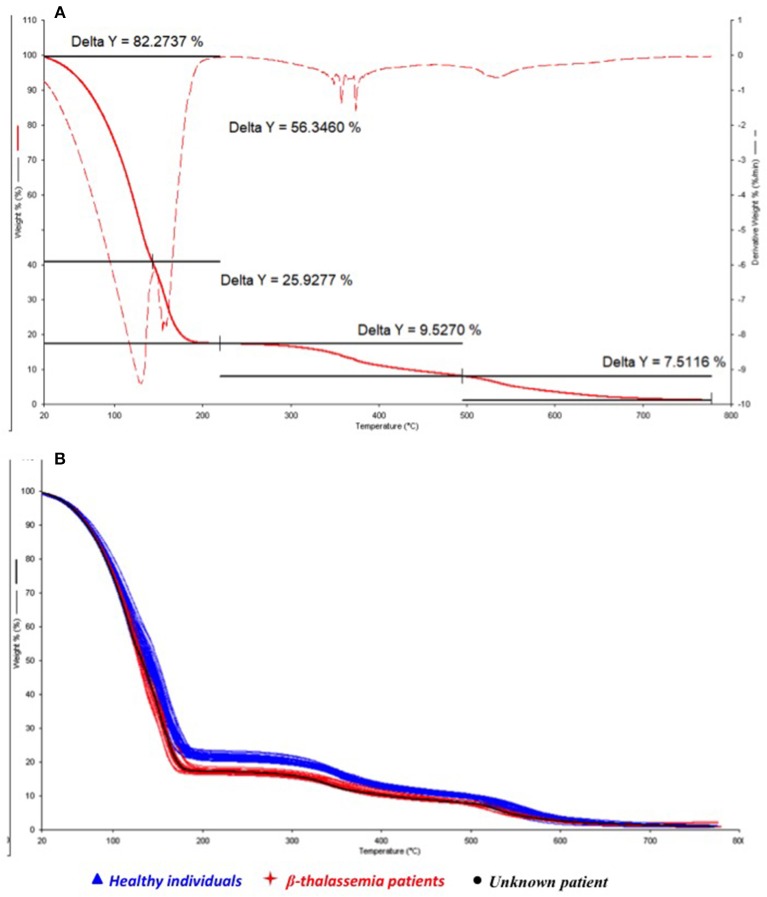
Overlapped thermogravimetric (TG) and Derivative Thermogravimetric (DTG) curves of the hemolytic anemia patient **(A)** and comparison **(B)** of the TG profile of the hemolytic anemia patient (black) with healthy subjects (blue) and thalassemic patients (red).

In order to search for an explanation of the hemolytic anemia and to verify the diagnostic power of the TGA/chemometric screening test, the thermal behavior of the unknown blood sample was estimated by thermogravimetry (Figure 1A, solid line) and the characteristic thermally induced decomposition processes under combustive conditions were investigated by calculating the derivative thermogravimetric curve (DTG) of the TG (Figure 1A, dashed line).

In accordance with previously findings (Risoluti et al., 2016b), three main releasing steps can be observed in Figure 1A, corresponding to the loss of water (occurring between 50 and 200°C) and the decomposition of the corpuscular fraction of blood (around 350 and 550°C). Two different processes may be described during water release: the first, at lower temperatures (20–130°C) is related to the bulk water release (56.3%) while the second peak (130–180°C) may be attributed to the bound water release (25.9%). The remaining processes lead to a percentage weight losses of 9.5 and 7.5% with a final residue close to zero.

The thermal profile of the patient's samples (black line) was consequently compared to those observed for the healthy and thalassemia subjects, respectively, reported in blue and red in Figure 1B, where the TG curves are overlapped. Results of the integration of the decomposition processes for all the investigated samples are reported in Table 2.

**Table 2:** Thermogravimetric features of the hemolytic anemia subject (patient) in comparison with β-Thalassemia patients (T) and healthy subjects (CTR) groups.

	**Water content (%)**	**Bulk water (%)**	**Bound water (%)**	**Bulk/bound water ratio**	**2nd weight loss (%)**	**3rd weight loss (%)**
CTR	77.7 ± 0.9	48.4 ± 5.3	29.3 ± 4.0	1.7 ± 0.6	11.4 ± 0.7	9.2 ± 0.6
T	82.5 ± 1.2	57.3 ± 6.5	25.2 ± 5.9	2.5 ± 1.0	9.0 ± 0.7	6.9 ± 0.7
*p*-value	1.5E–17	2.3E–05	2.0E–02	2.2E–02	4.7E–12	2.6E–15
Patient	82.5 ± 0.1	51.9 ± 2.6	30.9 ± 2.8	1.8 ± 0.4	8.6 ± 0.1	6.5 ± 0.2

A significant lower amount of water content (*p*-value of 1.5 E-17) was observed in healthy subject with respect to thalassemic ones, and a consequent lower value of the bulk/bound water ratio. On the contrary, the corpuscular fraction of blood was found to be higher in healthy subjects than thalassemia patients. The thermogravimetric profile of the patient's blood (Figure 1) was very distinct from those of healthy subjects and comparable with that of thalassemia subjects. In particular, the water amount of the patient was found to be within the group of thalassemics, as well as the decomposition processes of the corpuscular fraction.

**Figure 2:**
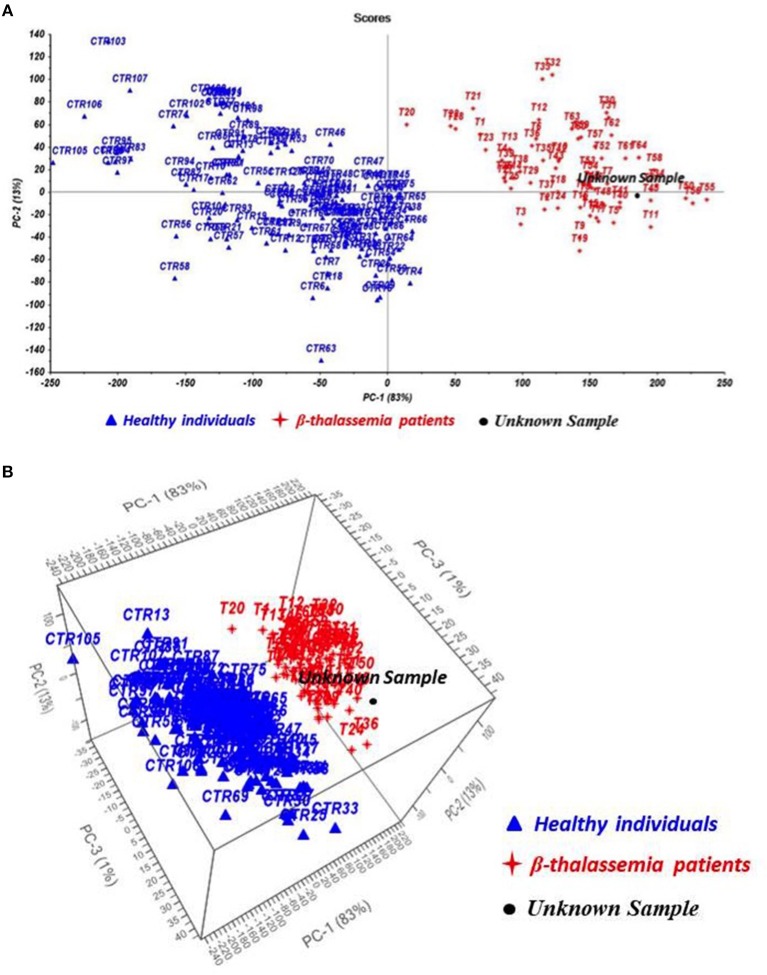
Chemometric outcomes from Principal Component Analysis (PCA) **(A)** and Partial Least Square Discriminant Analysis (PLS-DA) **(B)** of healthy subjects (CTR, blue), thalassemic patients (T, red), and hemolytic anemia patient (unknown sample, black).

The acquired TG curves were processed by the novel test TGA/Chemometrics and Principal Component Analysis algorithm was used to display results. In Figure 2A, the resulting scores plot exhibits a significant separation of the samples according to the presence or the absence of the anemic traits, resulting in two clusters of samples, the healthy subjects (blue) and the thalassemia patients (red), located in different side of the plot.

The classification model applied to the TG curve of the patient identified a chronic hemolytic anemia and in particular, the sample was located in the group of thalassemia patients suggesting the presence of a hemoglobin defect (Figure 2B).

### Molecular Analysis of the Globin Genes

To confirm the results obtained by TGA/chemometrics test the presence of a hemoglobin defect was investigated by molecular analysis of the globin genes. The molecular characterization of α and β globin genes, demonstrated the presence of a very rare hemoglobin variant Hb Bibba (α_2_136(H19)Leu → Proβ_2_) due to the substitution of a leucine residue in position 136 of the alpha chain by a proline residue. This α chain abnormal hemoglobin is an unstable hemoglobin with an autosomal dominant inheritance and was the cause of the chronic hemolytic anemia. Hb Bibba is an uncommon unstable hemoglobin variant which was not detected by the common protocols for the diagnosis of hemoglobinopathies. Therefore, the molecular analysis confirmed the results of the TGA/chemometrics test and demonstrated the capability of this method to discriminate healthy subjects and patients with a rare hemoglobin variant Hb Bibba not diagnosed by the common screening tests.

## Discussion

The TGA/Chemometric approach, previously used for diagnosis of thalassemia (Risoluti et al., 2016b, 2018b), was applied for the first time to investigate a rare case of chronic hemolytic anemia of difficult interpretation, for which the first level tests of the conventional diagnostic protocols were not able to find the erythrocyte congenital defect. The TGA/chemometric screening test allowed to make diagnosis of hemoglobinopathy, which was confirmed by the second level tests. The molecular analysis of the globin genes demonstrated the presence of a rare hemoglobin variant Hb Bibba (α_2_136(H19)Leu → Proβ_2_) which was the cause of the chronic hemolytic anemia.

In this study, a new method to obtain an early detection of hemoglobinopathy by a TGA/chemometric screening test is proposed, a method that requires few microliters of blood sample that are directly analyzed without any pre-treatment. This method results particularly suitable in pediatric patients as it requires small sample volumes and is able to detect hemoglobinopathies also in transfused patients (Risoluti et al., 2016b). Our results demonstrate that this diagnostic approach permits the screening of hemoglobinopathies in patients with heterogeneous clinical phenotype, as in this case of chronic hemolytic anemia characterized by microcytosis, hypochromia, normal HbA_2_, and HbF that was not diagnosed by the common first level protocols of investigation for congenital hemolytic anemias.

TGA/chemometric screening test is able to detect hemoglobinopathies determined by both quantitative defects as thalassemias and qualitative defects due to structural hemoglobin variants and not only in presence of hypochromic and microcytic anemia, but also in macrocytic anemia, and conditions that need molecular analysis for diagnosis such as δβ-thalassemia and β-thalassemia combined with Hb Lepore (Risoluti et al., 2016b, 2018a,b, 2019).

The effectiveness of this approach mainly consists of the multiparametric evaluation of the blood samples during the thermally induced decomposition under controlled temperature scanning. In fact, chemometric tools permit to correlate the entire TG curve of the anemic patient, to the collected dataset and to recognize a fingerprint profile of anemia.

## Conclusions

The TGA/Chemometrics test is a new screening method for an early detection not only of thalassemia but also hemoglobin defects. The ability of the TGA/Chemometrics method to early detect an anemic status due to an unstable hemoglobin variant is very attractive, so this new method could provide a new approach for diagnosis of congenital hemolytic anemias. The positive outcome to this test would permit to immediately address patients to confirmatory analyses for hemoglobinopathies with a consequent save in time and costs and to quickly identify the correct therapeutic protocol for the patient.

## Data Availability Statement

The datasets generated for this study are available on request to the corresponding author.

## Ethics Statement

The studies involving human participants were reviewed and approved by Comitato Etico Roma 2 of the S. Eugenio Hospital, Rome. Written informed consent for participating to the study and publishing clinical data in an anonymized manner are collected and copies of the informed consent are available on request. In the case of child, written informed consent for participating to the study and publishing clinical data in an anonymized manner was obtained from the parents.

## Author Contributions

RR, SM, and PC conceived the study and wrote the manuscript. Data analysis was performed by contributions of all authors. All authors have approved the final version of the manuscript.

### Conflict of Interest

The authors declare that the research was conducted in the absence of any commercial or financial relationships that could be construed as a potential conflict of interest.
